# Haemoglobin levels are associated with echocardiographic measures in a Finnish midlife population

**DOI:** 10.1080/07853890.2024.2425061

**Published:** 2024-12-03

**Authors:** Joona Tapio, Tommi Grönlund, Kari Kaikkonen, M. Juhani Junttila, Mikko P. Tulppo, Peppi Koivunen

**Affiliations:** a Biocenter Oulu and Faculty of Biochemistry and Molecular Medicine, Oulu Center for Cell-Matrix Research, University of Oulu, Oulu, Finland; b Research Unit of Biomedicine and Internal Medicine, University of Oulu, Oulu, Finland; c Medical Research Center Oulu, Oulu University Hospital and University of Oulu, Oulu, Finland

**Keywords:** Cardiovascular disease, echocardiography, global longitudinal strain, haemoglobin

## Abstract

**Background:**

Within normal variation, higher haemoglobin (Hb) levels are associated with unhealthier body composition, adverse metabolism and cardiovascular disease (CVD)-related mortality. Global longitudinal strain (GLS) is a direct, well validated and reproducible echocardiographic measure for the evaluation of cardiac contractile function, providing additional prognostic value for prediction of a variety of cardiac events. This study investigated the relation between Hb levels and cardiac function measures, including GLS, in a Finnish midlife population.

**Materials and methods:**

Echocardiography was carried out in a subpopulation of the Northern Finland Birth Cohort 1966 at age of 46 (*n* = 1155). GLS was available for *n* = 796. Subjects with diabetes, severe cardiac diseases, echocardiographic abnormalities, heart rate ≥85 bpm during echocardiography or Hb level outside the Finnish reference intervals (117–155 g/L for females and 134–167 g/L for males) were excluded from the analysis. The study population included 635 subjects (46% males). The data were analysed in Hb tertiles and in multivariable linear regression models.

**Results:**

The highest Hb tertile had adverse anthropometric and metabolic parameters compared to other Hb tertiles. Of the studied echocardiographic parameters, the highest Hb tertile had the highest left ventricular mass (LVM, *p* < .01), LVM index (LVMi, *p* < .05), LV end-diastolic volume (LVEDV, *p* < .05), posterior wall thickness (PWT, *p* < .001), relative wall thickness (RWT, *p* < .05) and the lowest absolute GLS (*p* < .001) but no difference in LV ejection fraction (LVEF) was observed between the Hb tertiles. In linear models, when adjusted for covariates, Hb levels were associated positively and independently with GLS (*B* = 0.153 [0.071; 0.235]) and LVM (*B* = 0.272 [0.193; 0.350]).

**Conclusions:**

Higher Hb levels are associated with an adverse metabolic and inflammatory profile and more adverse cardiac function measures, including GLS, in both sexes in midlife.

## Introduction

Cardiovascular diseases (CVDs) are a leading cause of death and disability, responsible for 32% of all deaths [1]. Left ventricular dysfunction (LVD) is more frequent among subjects with a diagnosis of heart disease and has predictive value for CVDs even when not associated with a definite diagnosis of a heart disease [2–4]. Clinical classification of patients with LVD can be evaluated using echocardiographic parameters such as the left ventricular ejection fraction (LVEF). Methods based on deformation measurements have been shown to identify early myocardial dysfunction before LVEF decrease [5]. Global longitudinal strain (GLS), which expresses longitudinal shortening of the heart as a percentage, is a direct, well validated and reproducible method for the evaluation of cardiac contractile function [6]. Prior studies have shown GLS to be more sensitive in evaluating LVD than LVEF and to provide additional prognostic value for predicting a variety of cardiac events and morbidities [7–9].

Haemoglobin (Hb) is the main carrier of oxygen in the cardiovascular system. Hb levels are regulated genetically and environmentally, and they vary by sex, race, age, altitude and acquired factors such as smoking [10]. In general, high-end Hb levels within the normal range are considered beneficial for health. Extremely high or low Hb levels and high Hb levels within a normal variation range have been reported as predictors of total and CVD-related mortality [11–13]. Multiple studies with selected cohorts have shown associations of higher Hb levels with individual co-morbidities of CVDs, including diabetes, dyslipidaemia and hypertension [14–17]. Hyperviscosity or changes in plasma volume [14–16], endothelial cell dysfunction [15] or higher iron/ferritin levels [17] have been suggested as mediators of these associations. However, we have recently shown that within normal reference interval, lower Hb levels associate with lower body mass index (BMI) and with an overall healthier metabolic profile both in males and females in middle age Finnish cohorts and that these alterations are mediated by relative tissue hypoxia and activation of the hypoxia-inducible factor (HIF) response in comparison to higher Hb levels [18].

Anaemia and iron deficiency are well-known to be associated with echocardiographic measures in patients with heart failure, influencing the symptoms and prognosis of the disease [19]. However, analyses on the relation of echocardiographic measures and Hb levels in the general population are lacking. The aim of this study was to assess the association of Hb levels within normal range with cardiac function measures including GLS, in a middle-aged Finnish population cohort.

## Materials and methods

### Study population

A flowchart of the study population is presented in Figure S1 in the supplemental material. Study population is a subpopulation of the general population-based longitudinal birth cohort, the Northern Finland Birth Cohort 1966 (NFBC1966) [20,21] (Figure S1). The NFBC1966 is a prospective general population-based longitudinal birth cohort, including 12,058 subjects (96.3% of all live births during the year 1966 in the two northernmost provinces of Finland). The data collection started in 1965 when the mothers were pregnant and so far, data have been collected at 1, 14, 31 and 46 years of age. Detailed information about the NFBC1966 has been previously described [20,21]. At age 46 years, a total of 10,282 subjects (85.2%) with known addresses in Finland were invited for clinical examination. Of the invited subjects, 5861 (57%) participated in the clinical examinations. Of the participants in clinical examinations, echocardiography was conducted for a randomly selected subpopulation of 1155 (19.7%).

For the current study, participants with a suboptimal quality of echocardiography (*n* = 359), heart rate ≥85 bpm during echocardiography (*n* = 65), any significant structural or functional cardiac pathologies (*n* = 30) or diagnosis of diabetes (*n* = 31) were excluded. The diagnosis of diabetes was made in line with the criteria of the World Health Organization (WHO) [22]: fasting blood glucose (fB-glucose) ≥7.0 mmol/L, or two-hour glucose in oral glucose test ≥11.0 mmol/L or glycated Hb ≥6.5%. Patients self-reported their medical history and subjects with severe cardiac defects were excluded. Severe defects included congenital heart disease, severe heart failure and severe coronary artery disease. Inclusion criteria for Hb levels were Hb levels within the Finnish reference values (117–155 g/L for women and 134–167 g/L for men). The Finnish reference values for Hb represent the age-correlated 2.5–97.5% Hb reference range determined by the Finnish National Working Group for basic blood count reference intervals [23]. The final study population included 635 subjects (289 males and 346 females).

For analyses, the study population was divided into sex-specific tertiles according to Hb levels (Figure S1). The corresponding sex-specific tertiles were then pooled to form Hb tertiles (low, medium and high) each of which consisted of the corresponding sex-specific tertiles from both sexes (Figure S1).

The authors obtained permission from the dataset owner, NFBC, to use the information for the purposes of the study. The NFBC1966 study was conducted according to the Declaration of Helsinki and approved by the Ethical Committee of Northern Ostrobothnia Hospital District in Oulu, Finland. All participants of NFBC1966 provided written informed consent. Analyses of the current study were conducted retrospectively and approved by the NFBC project center under project number P1071.

### Echocardiographic measurements

An experienced cardiologist (K.K.) performed a comprehensive transthoracic two-dimensional echocardiography using a General Electric Vivid E9 device with an M5S-D 1.5/4.6 MHz sector transducer for cardiovascular imaging (GE Health Medical, Horten, Norway). The measurements were performed by the guidelines of the American Society of Echocardiography [24]. In assessment of cardiac structure and function, we focused on the following echocardiographic variables: left ventricular mass (LVM), left ventricle end-diastolic volume (LVEDV), interventricular septal thickness (ST), posterior wall thickness (PWT), relative wall thickness (RWT), left atrium end-systolic volume (LAESV), LVEF and the ratio of early diastolic mitral inflow velocity to early diastolic mitral annulus velocity as a marker of diastolic function (*E*/*e*′). As a marker of systolic function, GLS was later calculated using Echopac 7 software (automated function imaging). For GLS values, the values given and used in the analyses are exact. Absolute value describes the exact values’ distance to zero. RWT was calculated as two times PWT divided by left ventricle (LV) diastolic diameter [24]. Measures of the cardiac structure were indexed to body surface area (the Dubois equation).

### Lifestyle parameters

Participants self-reported their smoking habits. Smoking status was categorized as never, former or current smokers. Insufficient sleep was determined by a questionnaire. Asking if subjects typically felt tired in the morning within 30 min of waking up, answers ‘very tired’ or ‘somewhat tired’ were defined as insufficient, and ‘somewhat rested’ or ‘well-rested’ were defined as sufficient sleep [25]. Total sitting time was estimated based on self-reported sitting hours during weekdays (at work, home, commuting/in a vehicle, or elsewhere) [25]. Total sum of sitting time was then dichotomized using a previously established cutoff value of 11 h/d [26]. Usage of antihypertensive or lipid-lowering medication was determined by a questionnaire and patient records. Daily alcohol consumption was estimated based on the self-reported frequency and number of alcoholic beverages consumed [25]. The subjects were categorized into three groups according to Finnish Institute for Health and Welfare (THL) alcohol consumption risk levels [27]. The cut-off values were: 10 g/d (moderate) and 20 g/d (high) for women, and 20 g/d (moderate) and 40 g/d (high) for men [25]. Physical activity was measured using a wrist-worn, waterproof accelerometer (Polar active, Polar Electro Oy, Kempele, Finland). The participants were asked to wear the accelerometer 24 h a day for at least 14 days on the wrist of their non-dominant hand. The device collects physical activity data and presents it as metabolic equivalent (MET) values for every 30 s. Intensity levels were provided by the manufacturer and the daily averages spent in the following activity levels were calculated: 1.00–1.99 MET = sedentary, 2.00–3.49 MET = light, 3.50–4.99 MET = moderate, 5.00–7.99 = vigorous and ≥8.00 = very vigorous [28]. The accelerometers were blinded, thus giving no feedback to the subjects [28]. The daily amount of moderate to vigorous physical activity in minutes (MVPA) was used in the analyses.

### Anthropometric and clinical measurements

Body weight was measured with a regularly calibrated digital scale. Height was measured twice using a standard and calibrated stadiometre and the mean of the two measurements was used. BMI was calculated as the ratio of weight (kg) and height squared (m^2^). Waist and hip circumferences were measured twice and mean of the two measurements was used. Waist–hip ratio (WH ratio) was assessed as the ratio between circumferences of the waist (at the level midway between lowest rib margin and the iliac crest) and the hip (at the widest trochanters). All anthropometric measurements were done after overnight (12 h) fasting period.

Brachial systolic blood pressure (sbp) and diastolic blood pressure (dbp) were measured three times with 1 min interval after 15 min of rest from the right arm of the seated participants using an automated oscillometric blood pressure device and appropriately sized cuff (Omron Digital Automatic Blood Pressure Monitor Model M10-IT, Kyoto, Japan). Finally, the mean of two lowest systolic values and their diastolic values was used in the analyses. Mean arterial pressure (MAP) was calculated using the following formula:

$$\text{MAP}=\frac{\left[\text{sbp}+\left(2\times \text{dbp}\right)\right]}{3}.$$

### Laboratory measures and derivatives

Blood samples of NFBC1966 were taken after an overnight fasting period and centrifuged and analysed immediately without storing. NFBC1966 blood samples were analysed in NordLab Oulu (former name Oulu University Hospital, Laboratory), a testing laboratory (T113) accredited by Finnish Accreditation Service (FINAS) (EN ISO 15189). Fasting blood glucose and fasting serum insulin (fs-insulin) levels of NFBC1966 were determined by radioimmunoassay (Pharmacia Diagnostics, Uppsala, Sweden) and analysed by an enzymatic dehydrogenase method (Advia 1800, Siemens Healthcare Diagnostics, Tarrytown, NY) and by a chemiluminometric immunoassay (Advia Centaur XP, Siemens Healthcare Diagnostics, Tarrytown, NY), respectively. To evaluate insulin resistance, homeostatic model assessment of insulin resistance (HOMA-IR) was calculated accordingly (fB-glucose × fs-insulin/22.5). HbA1c was analysed at the cohort laboratory between 7:00 AM and 11:00 AM after an overnight fasting period using immunochemical assay methods (all methods by Advia 1800; Siemens Healthcare Diagnostics Inc., Tarrytown, NY). Total, high-density lipoprotein (HDL) and low-density lipoprotein (LDL) cholesterol levels and serum triglycerides were determined using an enzymatic assay method (Advia 1800; Siemens Healthcare Diagnostics Inc., Tarrytown, NY). Blood Hb and red cell parameters including mean cellular volume (MCV), red cell distribution width (RDW), mean cellular Hb (MCH) and mean cellular Hb concentration (MCHC) were determined using a Sysmex XE-2100 analyser (Sysmex Corporation, Kobe, Japan). The whole blood Hb levels of NFBC1966 were determined using spectrophotometric methods, red blood cells (RBCs) were measured using electric resistance detecting methods (impedance technology) with hydrodynamic focusing. Haematocrit (HCT) was determined by applying RBC pulse-height detection. Complete blood cell count was done on fresh plasma samples using automatic electronic cell counter (Coulter Corp., Miami, FL).

### Statistical analyses

The data were analysed using SPSS software (IBM SPSS Statistics 28; IBM Corp, Armonk, NY). The dependent variables were checked for normality (Gaussian distribution) by visual inspection. One-way ANOVA was used for tertile comparisons. Bonferroni’s *post hoc* test was used when applicable. In the tertile analyses, a *p* value of <.05 was considered statistically significant. *p* Values < .001 were not given as exact values. No significant interaction between the sexes was found, so men and women were analysed conjointly. Multivariate linear regression analysis (enter method) was used to estimate the relationship between the response variables and explanatory variables and to assess the contribution of potential confounders. The following variables and models were considered: sex (model 1), the Framingham risk factors (total cholesterol, HDL cholesterol, smoking habits and sbp) (model 2), and WH ratio, dbp and fB-glucose (model 3). Model 3 was chosen based on a stepwise analysis to identify the most important predictor variables of GLS. *B* values were given as standardized values. The confidence intervals for *B* weights were estimated by running multiple regression analyses with *Z*-score standardized predictor variables.

## Results

### Characteristics of the study population in Hb tertiles

Characteristics of the study population (grouped by Hb tertiles) are presented in Table 1. All Hb tertiles; low, medium and high, had a similar number of male subjects (Table 1). Significant differences in several characteristics between the Hb tertiles were observed (Table 1). No difference in smoking, sleeping habits, time spent sitting, usage of lipid-lowering or antihypertensive medication, alcohol consumption or MVPA was observed between the Hb tertiles (Table 1). Subjects in the high Hb tertile had more adverse anthropometric measures, the highest bp values, more adverse glucose values including the highest insulin resistance scores and the unhealthiest lipid profile compared to subjects in other Hb tertiles (Table 1). In line with having the highest Hb levels, the subjects in the high Hb tertile also had the highest HCT, RBC count, MCH and MCHC (Table 1). Subjects in the low Hb tertile had the highest RDW (Table 1). No difference in platelet count or MCV was observed between the Hb tertiles (Table 1). Subjects in the high Hb tertile had the highest total leucocyte count including the highest blood neutrophil, lymphocyte and monocyte counts, while no difference in blood eosinophil or basophil counts were observed (Table 1).

**Table 1.** Characteristics of the study population in Hb tertiles.

Variable	Low Hb tertile	Medium Hb tertile	High Hb tertile	*p* Value
Hb (g/dL)	132.3 (9.1)	139.9 (8.1)	151.0 (8.6)	<.001
Number of subjects (*n*)	212	210	213	
Number of males (*n*)	96	86	107	.159
Current smokers (*n*)	47	44	49	.890
Insufficient sleep (*n*)	65	67	59	.670
Sitting time ≥11 h/d (*n*)	27	40	27	.105
Bp medication (*n*)	28	25	20	.455
Lipid medication (*n*)	2	6	5	.355
Alcohol consumption (g/d)	9.8 (17.1)	10.6 (17.3)	11.4 (19.8)	.649
MVPA (min/d)	73.1 (33.0)	70.1 (36.1)	71.3 (32.8)	.673
Height (cm)	171.4 (8.9)	171.1 (9.3)	172.1 (8.5)	.490
Weight (kg)	73.3 (12.3)	75.0 (14.0)	81.2 (14.5)	<.001
BMI (kg/m^2^)	24.8 (3.0)	25.5 (3.8)	27.4 (4.3)	<.001
WH ratio	0.89 (0.07)	0.89 (0.08)	0.91 (0.08)	<.001
Sbp mean (mmHg)	120.9 (13.5)	122.4 (14.2)	126.5 (17.6)	<.001
Dbp mean (mmHg)	80.5 (9.2)	82.7 (9.8)	85.1 (10.6)	<.001
MAP (mmHg)	94.0 (10.3)	95.9 (10.9)	98.9 (12.5)	<.001
fB-glucose (mmol/L)	5.3 (0.5)	5.3 (0.4)	5.4 (0.5)	<.001
HOMA-IR	1.44 (1.23)	1.59 (1.54)	2.2 (1.71)	<.001
Total cholesterol (mmol/L)	5.3 (0.9)	5.3 (0.9)	5.4 (0.9)	.210
HDL cholesterol (mmol/L)	1.60 (0.37)	1.59 (0.38)	1.49 (0.34)	.004
LDL cholesterol (mmol/L)	3.3 (0.9)	3.4 (0.9)	3.6 (0.9)	.004
Triglycerides (mmol/L)	0.95 (0.54)	1.01 (0.67)	1.08 (0.72)	<.001
Hematocrit (%)	0.40 (0.02)	0.42 (0.02)	0.44 (0.02)	<.001
RBC count (×10^12^/L)	4.4 (0.3)	4.6 (0.3)	4.9 (0.3)	<.001
MCV (fL)	89.6 (3.8)	90.0 (3.4)	90.4 (3.5)	.072
MCH (pg)	30.0 (1.5)	30.3 (1.3)	30.8 (1.2)	<.001
MCHC (g/dL)	334 (8)	337 (7)	340 (7)	<.001
RDW (%)	13.4 (0.8)	13.1 (0.6)	13.1 (0.6)	<.001
B-platelets (×10^9^/L)	254 (55)	248 (56)	248 (49)	.470
B-leucocytes (×10^9^/L)	5.0 (1.22)	5.3 (1.2)	5.6 (1.5)	<.001
B-neutrophils (×10^9^/L)	2.8 (1.0)	2.9 (0.9)	3.2 (1.1)	<.001
B-lymphocytes (×10^9^/L)	1.6 (0.5)	1.7 (0.5)	1.8 (0.49)	.001
B-monocytes (×10^9^/L)	0.45 (0.13)	0.46 (0.17)	0.50 (0.17)	.001
B-eosinophils (×10^9^/L)	0.19 (0.13)	0.20 (0.13)	0.20 (0.14)	.771
B-basophils (×10^9^/L)	0.03 (0.02)	0.03 (0.02)	0.03 (0.02)	.260

Hb: haemoglobin; bp: blood pressure; MVPA: moderate to vigorous physical activity; BMI: body mass index; WH: waist–hip; Sbp: systolic blood pressure; Dbp: diastolic blood pressure; MAP: mean arterial pressure; fB-glucose: fasting blood glucose; HOMA-IR: homeostatic model assessment for insulin resistance; HDL: high-density lipoprotein; LDL: low-density lipoprotein; RBC: red blood cell; MCV: mean cellular volume; MCH: mean cellular haemoglobin; MCHC: mean cellular haemoglobin concentration; RDW: red cell distribution width; B: blood.

The values indicate exact numbers of study subjects, mean (SD, standard deviation) or median (inter quartile range). *p* is given for statistical comparison of low and High Hb tertiles. Number of participants (*n*) in the statistical analyses.

Similar differences were observed in sex-specific Hb tertile analyses for most parameters (Tables S1 and S2). However, the differences in lipid profile were only observed in females and this was also the case for MCV, RDW, neutrophil, lymphocyte and monocyte counts (Tables S1 and S2).

### Echocardiographic characteristics in Hb tertiles

Echocardiographic characteristics of the study population in Hb tertiles are presented in Table 2. No difference in resting heart rate was observed between the Hb tertiles (Table 2). The high Hb tertile had the highest LVM, LVM index (LVMi) and LVEDV (Table 2). No significant difference in LVEDV index (LVEDVi) or ST at diastole or its index (STi) was observed between the Hb tertiles (Table 2). Subjects in the high Hb tertile had the highest PWT while its index (PWTi) showed no difference between the Hb tertiles (Table 2). The high Hb tertile had the highest RWT (Table 2). No difference in LAESV, its index (LAESVi) or LVEF biplane was observed between the Hb tertiles (Table 2). Subjects in the high Hb tertile had the lowest absolute GLS while no difference was observed in *E*/*e*′ (Table 2).

**Table 2.** Echocardiography characteristics in the study population.

Variable	Low Hb tertile	Medium Hb tertile	High Hb tertile	*p* Value
Number of subjects (*n*)	212	210	213	
Heart rate rest (bpm)	66 (10)	67 (10)	67 (9)	.156
LVM (g)	169.2 (47.0)	172.1 (50.0)	185.9 (55.9)	.002
LVMi (g/m^2^)	90.2 (19.6)	91.2 (19.7)	95.4 (23.0)	.026
LVEDV (mL)	97.7 (24.8)	97.4 (24.5)	103.4 (27.6)	.027
LVEDVi (mL/m^2^)	53.1 (10.6)	52.1 (9.8)	52.0 (10.6)	.450
ST at diastole (cm)	0.92 (0.17)	0.94 (0.16)	0.95 (0.17)	.083
STi (cm/m^2^)	0.49 (0.07)	0.50 (0.07)	0.49 (0.08)	.282
PWT (cm)	0.88 (0.14)	0.89 (0.15)	0.93 (0.15)	<.001
PWTi (cm/m^2^)	0.47 (0.06)	0.48 (0.06)	0.48 (0.07)	.322
RWT	0.34 (0.06)	0.35 (0.06)	0.36 (0.06)	.027
LAESV (mL)	55.1 (15.9)	54.3 (16.9)	55.5 (15.9)	.739
LAESVi (mL/m^2^)	29.7 (7.6)	28.9 (7.5)	28.6 (7.0)	.322
LVEF biplane (%)	61.1 (5.5)	60.7 (5.9)	60.9 (6.2)	.811
GLS (%)	−21.3 (2.3)	−20.9 (2.6)	−20.2 (2.7)	<.001
*E*/*e*′	7.1 (1.5)	7.2 (1.6)	7.3 (1.6)	.376

LVM: left ventricular mass; i: index; LVEDV: left ventricular end-diastolic volume; ST: septal thickness: PWT: posterior wall thickness; RWT: relative wall thickness; LAESV: left atrial end-systolic volume; LVEF: left ventricular ejection fraction; GLS: global longitudinal strain; *E*/*e*′: ratio of early diastolic mitral inflow velocity to early diastolic mitral annulus velocity.

The values indicate exact numbers of study subjects, mean (SD, standard deviation) or median (inter quartile range) for the variables used in the analysis. *p* is given for statistical comparison of low and high Hb tertiles. Number of participants (*n*) in the statistical analyses.

In sex-specific analyses, significant differences between the Hb tertiles in LVM, LVEDV, ST at diastole, PWT and RWT were only observed for females while the difference in absolute GLS was similar for both sexes (Tables S3 and S4).

### Association of Hb levels with selected echocardiographic parameters

**Figure 1.**
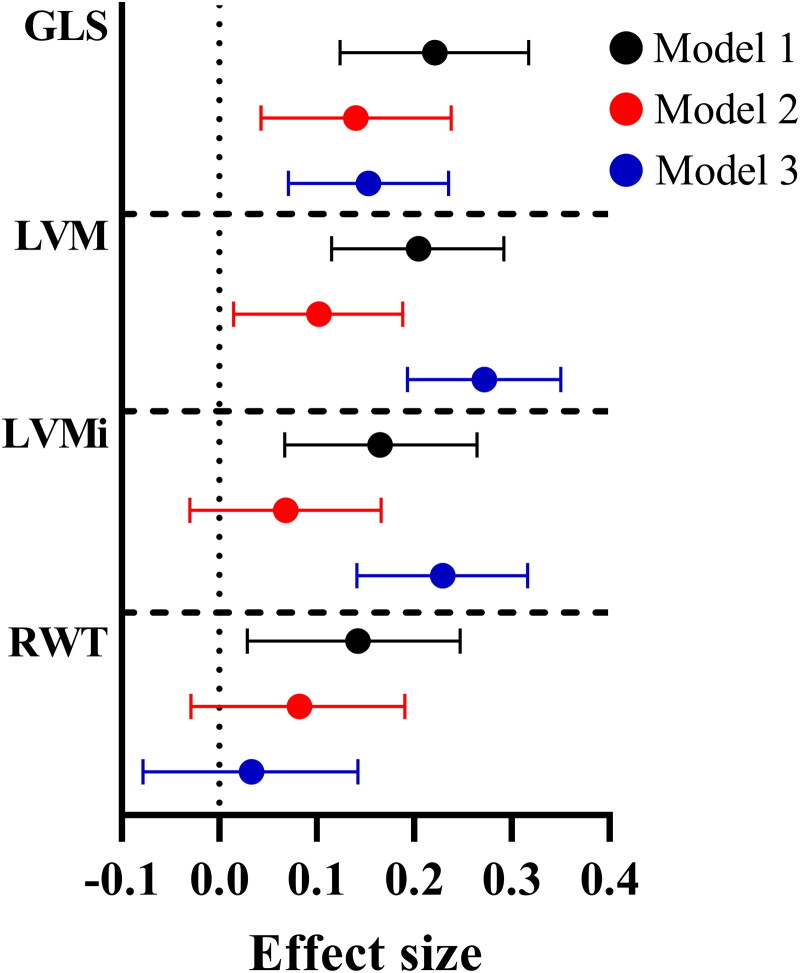
Effect sizes for association of Hb levels with echocardiographic parameters. Forest plot representing the standardized effect sizes and their 95% confidence intervals for selected echocardiographic parameters. GLS: global longitudinal strain; LVM: left ventricular mass; i: index; RWT: relative wall thickness. Model 1 (black) is adjusted for sex. Model 2 (red) is adjusted for Framingham risk factors (sex, body mass index, systolic blood pressure, smoking status, total cholesterol and HDL cholesterol). Model 3 (blue) is a stepwise linear regression adjusted for fasting glucose, diastolic blood pressure and waist–hip ratio.

Three linear models of association of Hb levels and selected echocardiographic parameters are presented in Figure 1. Model 1 (black) was adjusted for sex, model 2 (red) for the Framingham risk factors (sex, BMI, sbp, smoking status, total cholesterol and HDL cholesterol) and model 3 (blue) shows a stepwise analysis to identify the most important predicting variables of absolute GLS and which was adjusted for fB-glucose, dbp and WH ratio (Figure 1, Table S5). In all three models, Hb levels associated positively with absolute GLS and LVM (Figure 1, Table S5). Positive association of Hb levels with LVMi in models 1 and 3 and RWT in model 1 were also observed (Figure 1, Table S5).

In sex-specific analyses, some differences were observed between males and females (Figure S2, Tables S6 and S7). Hb levels associated positively with absolute GLS in all three models in males but only in model 1 in females (Figure S2). For LVM, a positive association with Hb levels was observed in models 1 and 3 for males and in models 1 and 2 for females (Figure S2). For LVMi, a positive association with Hb levels was observed in model 3 for males while no association was observed for females (Figure S2). For RWT, no associations were observed for males while a positive association with Hb levels was observed in model 1 for females (Figure S2).

## Discussion

We have recently studied hypothesis-driven in a cohort-wide analysis, whether normal variation of Hb levels could be used as a surrogate measure for hypoxia [18]. We hypothesized that in comparison to higher Hb levels, lower Hb levels were hypoxic and could result via inhibition of the oxygen sensing HIF prolyl 4-hydoxylase (HIF-P4H) to HIF-mediated reprogramming of energy metabolism and better metabolic health, as we have earlier shown for mice with genetic or pharmacologic inhibition of HIF-P4Hs [29]. In the cohort-wide analysis of the NFBC1966, the studied metabolic parameters associated negatively with Hb levels at 31 and 46 years [18]. Lower Hb levels associated with higher expression of HIF target genes, such as glucose transporters, suggesting that Hb levels via regulation of tissue oxygenation can regulate energy metabolism via the HIF pathway, lower Hb levels being beneficial [18].

The aim here was to study the effect of Hb levels within normal range on cardiac parameters determined by echocardiography in human. We hypothesized that subjects with lower Hb levels would be protected from cardiac dysfunction, similarly to mice that have chronically active HIF response due to deficiency in the key HIF-P4H isoenzyme, HIF-P4H-2 or wild-type mice treated with a small molecule HIF-P4H inhibitor that are protected against atherosclerosis and age-induced cardiac hypertrophy [30,31]. The findings reported here are in line and extend previous results by us [13,18] and others [32,33], and are mostly associated with activation of HIF1α.

In the current study, we studied a subpopulation of the NFBC1966 birth cohort where a similar clustering of adverse metabolic parameters and higher Hb levels to the larger cohort were seen [18], although the current study included only some 20% of the whole study population having less statistical power. The results show that individuals with higher Hb levels possessed more adverse anthropometric, glucose metabolism and lipid profile parameters and higher bp values compared to subjects with lower Hb levels. From a clinical perspective, the low Hb tertile was the only one not classified as overweight with a mean BMI of 24.8. Although the differences between the Hb tertiles in bp values were minor, it is well-established that the risk for CVDs incrementally increases in relation to bp values [34].

Higher numbers of several but not all inflammatory cell lines were observed in the high Hb tertile compared to the others, the difference being more pronounced in females, suggesting an increased inflammatory load. No difference between platelet counts was detected in different Hb tertiles suggesting that the cell count differences were specific to RBCs and a subpopulation of leucocytes. HIF response is associated with inflammatory pathways, in general its activation being found as anti-inflammatory whereas in specific tissues it may have adverse effects [35].

Several differences in echocardiographic measures were observed between the Hb tertiles, some of these differences only reaching statistical significance in females. LVM, an indicator of LV hypertrophy, was the highest in the high Hb tertile compared to others in females, while only a trend of association was observed for males. Similar sex-associated differences were also observed in LVEDVi and PWT. However, for GLS the absolute value was the lowest in the high Hb tertile; the same being observed for both sexes. The statistical differences observed in the tertile analyses between males (*n* = 289) and females (*n* = 346) could be partly explained by more statistical power for females. In multivariable regression analyses, Hb levels associated positively with GLS and LVM in all three models, suggesting an independent association of Hb levels with these cardiac parameters. In sex-specific analyses, the association between Hb levels and GLS was stronger in males than females in all three models, while for LVM the strength of the association was more dependent on the model used.

The absolute value of GLS is decreased in impaired systolic deformation [36]. According to a meta-analysis, a normal absolute GLS at age of 46–50 years is defined as −21.2 ± 2.5. Accordingly, the here measured GLS values for all Hb tertiles were within the normal range [36]. In a follow-up study conducted in a Danish general population, GLS was shown to provide linear, independent and incremental prognostic information regarding long-term risk of CVD-related mortality, with the prognostic value being more pronounced in males [37]. This suggests that changes in GLS according to Hb levels observed here, even if small, could have clinical prognostic significance regarding CVDs and various cardiac diseases, as higher Hb levels are also an independent risk factor for adverse metabolism and CVD-related and total mortality [13]. Interestingly, although multiple associations between Hb levels and LV echocardiographic measurements were observed here, one was not observed between Hb levels and LVEF. This might be due to technical reasons. However, our findings support widespread utilization of GLS as an echocardiographic LV function measure, as GLS has previously been shown to have a superior prognostic value in evaluation of LVD over LVEF in a variety of cardiac diseases [7–9].

One study limitation is the use of Hb levels as a surrogate measure for oxygen levels without saturation data nor analyses of parameters affecting oxygen release and microvascular function. However, we have reported earlier in a subpopulation of NFBC1966 at age of 31 years an association of Hb levels with oxygen consumption [18]. Menstruation status is well-known to slightly affect Hb levels. A study conducted on 1369 Danish women aged 30–60 years reported average Hb levels of 137 g/L in premenopausal and 140 g/L in postmenopausal women [38]. Our analyses were carried out at perimenopausal age of 46 years. Since no hormonal measurements were available, we were not able to take in account the potential effect of menstruation status to our results. Although most data were adjusted for multiple covariates, residual confounding effects need to be considered as an explanatory factor. Also, we have not carried out correction for multiple testing. We believe, however, that in studies that are explanatory in nature, it is more important to report all significant findings, even if suggestive, to be considered for replication in another study than taking the risk of rejecting the positive results. Also, the current study presents no longitudinal data.

## Conclusions

The findings reported here show that higher Hb levels have predictive value for cardiac hypertrophy and sensitive changes to echocardiographic measures describing cardiac systolic function, such as absolute GLS, in a healthy middle age population. This may also be important in assessing long-term risks for CVD and mortality. Altogether, these data suggest that slight tissue hypoxia, for example by lower endogenous Hb levels, mediate many beneficial effects to cardiometabolic health.

## Supplementary Material

Table S6.docx

Table S7.docx

Table S4.docx

Table S3.docx

Table S2.docx

S2.tif

Table S1.docx

S1.tif

Clean copy - GLS_manuscript_SUPPLEMENTAL_MATERIAL.docx

Table S5.docx

## Data Availability

Restrictions apply to the availability of these data, which were used under license for this study. In the use of data, the NFBC follows the EU general data protection regulation (679/2016) and Finnish Data Protection Act. Data are available for research purposes via electronic material request portal (https://www.oulu.fi/en/university/faculties-and-units/faculty-medicine/northern-finland-birth-cohorts-and-arctic-biobank) with the permission of the NFBC project center (NFBCprojectcenter@oulu.fi) [21]. The use of personal data is based on a cohort participant’s written informed consent in their latest follow-up study.
