# Supplementary material for: Haemoglobin levels are associated with echocardiographic measures in a Finnish midlife population
Source: Ann Med. 2024 Dec 3;56(1):2425061. doi: 10.1080/07853890.2024.2425061 (PMC11616746; doi:10.1080/07853890.2024.2425061)
Supplement: Table S6.docx [file IANN_A_2425061_SM0715.docx]

| **Table S6 Effect sizes for association of Hb levels with echocardiographic parameters in males** | | | | | | |
| --- | --- | --- | --- | --- | --- | --- |
|  |  | **n** | **B** | **CIL** | **CIU** | ***P* value** |
| **GLS** | **Model 1** | 289 | 0.191 | 0.075 | 0.307 | < 0.001 |
|  | **Model 2** | 289 | 0.166 | 0.050 | 0.283 | 0.005 |
|  | **Model 3** | 289 | 0.117 | 0.005 | 0.229 | 0.041 |
| **LVM** | **Model 1** | 289 | 0.114 | 0.003 | 0.226 | 0.045 |
|  | **Model 2** | 289 | 0.089 | -0.024 | 0.201 | 0.123 |
|  | **Model 3** | 289 | 0.129 | 0.011 | 0.248 | 0.032 |
| **LVMi** | **Model 1** | 289 | 0.098 | -0.015 | 0.211 | 0.090 |
|  | **Model 2** | 289 | 0.086 | -0.029 | 0.201 | 0.143 |
|  | **Model 3** | 289 | 0.132 | 0.010 | 0.252 | 0.034 |
| **RWT** | **Model 1** | 289 | 0.077 | -0.042 | 0.199 | 0.201 |
|  | **Model 2** | 289 | 0.035 | -0.084 | 0.155 | 0.557 |
|  | **Model 3** | 289 | -0.021 | -0.137 | 0.095 | 0.719 |
