# Supplementary material for: Haemoglobin levels are associated with echocardiographic measures in a Finnish midlife population
Source: Ann Med. 2024 Dec 3;56(1):2425061. doi: 10.1080/07853890.2024.2425061 (PMC11616746; doi:10.1080/07853890.2024.2425061)
Supplement: Table S7.docx [file IANN_A_2425061_SM0714.docx]

| **Table S7 Effect sizes for association of Hb levels with echocardiographic parameters in females** | | | | | | |
| --- | --- | --- | --- | --- | --- | --- |
|  |  | **n** | **B** | **CIL** | **CIU** | ***P* value** |
| **GLS** | **Model 1** | 346 | 0.139 | 0.032 | 0.241 | 0.011 |
|  | **Model 2** | 346 | 0.069 | -0.034 | 0.171 | 0.192 |
|  | **Model 3** | 346 | 0.051 | -0.051 | 0.154 | 0.323 |
| **LVM** | **Model 1** | 346 | 0.177 | 0.070 | 0.285 | 0.001 |
|  | **Model 2** | 346 | 0.108 | 0.004 | 0.213 | 0.042 |
|  | **Model 3** | 346 | 0.089 | -0.013 | 0.193 | 0.088 |
| **LVMi** | **Model 1** | 346 | 0.098 | -0.011 | 0.206 | 0.078 |
|  | **Model 2** | 346 | 0.036 | -0.071 | 0.143 | 0.504 |
|  | **Model 3** | 346 | 0.036 | -0.072 | 0.144 | 0.509 |
| **RWT** | **Model 1** | 346 | 0.128 | 0.021 | 0.238 | 0.020 |
|  | **Model 2** | 346 | 0.083 | -0.025 | 0.193 | 0.131 |
|  | **Model 3** | 346 | 0.035 | -0.071 | 0.142 | 0.513 |
