# Supplementary material for: Haemoglobin levels are associated with echocardiographic measures in a Finnish midlife population
Source: Ann Med. 2024 Dec 3;56(1):2425061. doi: 10.1080/07853890.2024.2425061 (PMC11616746; doi:10.1080/07853890.2024.2425061)
Supplement: Table S4.docx [file IANN_A_2425061_SM0713.docx]

| **Table S4 Echocardiography characteristics of females in the study population** | | | | | |
| --- | --- | --- | --- | --- | --- |
| **Variable** | **All subjects** | **Low Hb** | **Medium Hb** | **High Hb** | ***P* value** |
| Number of subjects (n) | 346 | 116 | 124 | 106 |  |
| Heart rate rest (bpm) | 68.3 (9.4) | 67 (10) | 69 (9.3) | 68 (9) | 0.177 |
| LVM (g) | 147.4 (34.0) | 141.9 (30.5) | 144.4 (30.9) | 157 (38.9) | 0.002 |
| LVMi (g/m^2^) | 83.2 (16.1) | 81.9 (15.3) | 81.9 (14.5) | 86.2 (18.1) | 0.074 |
| LVEDV (mL) | 87.3 (18.1) | 84.5 (16.4) | 87.1 (16.3) | 90.5 (21.3) | 0.047 |
| LVEDVi (mL/m^2^) | 49.8 (8.7) | 50.3 (9.3) | 49.9 (7.8) | 49.0 (9.1) | 0.511 |
| ST at diastole (cm) | 0.86 (0.15) | 0.84 (0.15) | 0.87 (0.14) | 0.88 (0.15) | 0.040 |
| STi (cm/m^2^) | 0.49 (0.08) | 0.48 (0.08) | 0.50 (0.07) | 0.49 (0.08) | 0.334 |
| PWT (cm) | 0.84 (0.13) | 0.81 (0.11) | 0.83 (0.11) | 0.87 (0.14) | 0.004 |
| PWTi (cm/m^2^) | 0.47 (0.07) | 0.47 (0.06) | 0.47 (0.06) | 0.48 (0.07) | 0.653 |
| RWT | 0.34 (0.06) | 0.33 (0.05) | 0.34 (0.06) | 0.35 (0.07) | 0.066 |
| LAESV (mL) | 50.6 (14.0) | 50.5 (14.3) | 49.8 (13.4) | 51.6 (14.3) | 0.610 |
| LAESVi (mL/m^2^) | 28.6 (7.0) | 29.3 (7.5) | 28.2 (6.6) | 28.4 (7.0) | 0.495 |
| LVEF biplane (%) | 61.6 (5.6) | 61.7 (5.7) | 61.6 (5.4) | 61.3 (5.8) | 0.828 |
| GLS (%) | -21.9 (2.2) | -22.2 (1.9) | -22.0 (2.3) | -21.4 (2.3) | 0.008 |
| E/e’ | 7.4 (1.6) | 7.3 (1.5) | 7.4 (1.6) | 7.5 (1.7) | 0.459 |
