# Supplementary material for: Haemoglobin levels are associated with echocardiographic measures in a Finnish midlife population
Source: Ann Med. 2024 Dec 3;56(1):2425061. doi: 10.1080/07853890.2024.2425061 (PMC11616746; doi:10.1080/07853890.2024.2425061)
Supplement: Table S3.docx [file IANN_A_2425061_SM0712.docx]

| **Table S3 Echocardiographic characteristics of males in the study population** | | | | | |
| --- | --- | --- | --- | --- | --- |
| **Variable** | **All subjects** | **Low Hb** | **Medium Hb** | **High Hb** | ***P* value** |
| Number of subjects (n) | 289 | 96 | 86 | 107 |  |
| Heart rate rest (bpm) | 64.6 (9.8) | 64 (10) | 65 (10) | 65 (10) | 0.633 |
| LVM (g) | 209.1 (48.8) | 200.1 (42.6) | 211.9 (45.0) | 214.2 (55.8) | 0.131 |
| LVMi (g/m^2^) | 102.9 (21.0) | 100.0 (19.6) | 104.4 (18.6) | 104.4 (23.7) | 0.248 |
| LVEDV (mL) | 114.3 (26.0) | 113.7 (23.9) | 112.4 (26.8) | 116.3 (27.3) | 0.557 |
| LVEDVi (mL/m^2^) | 55.6 (11.3) | 56.5 (11.2) | 55.2 (11.5) | 55.0 (11.2) | 0.584 |
| ST at diastole (cm) | 1.01 (0.15) | 1.01 (0.15) | 1.03 (0.14) | 1.02 (0.17) | 0.646 |
| STi (cm/m^2^) | 0.50 (0.07) | 0.50 (0.07) | 0.51 (0.06) | 0.50 (0.07) | 0.494 |
| PWT (cm) | 0.97 (0.13) | 0.95 (0.13) | 0.98 (0.14) | 0.99 (0.13) | 0.060 |
| PWTi (cm/m^2^) | 0.48 (0.06) | 0.47 (0.06) | 0.49 (0.06) | 0.48 (0.06) | 0.320 |
| RWT | 0.36 (0.06) | 0.35 (0.06) | 0.37 (0.06) | 0.37 (0.06) | 0.353 |
| LAESV (mL) | 60.2 (17.2) | 60.8 (16.0) | 60.8 (19.3) | 59.2 (16.5) | 0.761 |
| LAESVi (mL/m^2^) | 29.6 (7.8) | 30.2 (7.8) | 29.9 (8.7) | 28.9 (7.1) | 0.441 |
| LVEF biplane (%) | 60.1 (6.1) | 60.3 (5.1) | 59.4 (6.4) | 60.5 (6.6) | 0.442 |
| GLS (%) | -19.5 (2.5) | -20.2 (2.3) | -19.4 (2.4) | -19.0 (2.5) | 0.002 |
| E/e’ | 6.9 (1.4) | 6.8 (1.3) | 6.9 (1.6) | 7.0 (1.4) | 0.674 |
