# Supplementary material for: Haemoglobin levels are associated with echocardiographic measures in a Finnish midlife population
Source: Ann Med. 2024 Dec 3;56(1):2425061. doi: 10.1080/07853890.2024.2425061 (PMC11616746; doi:10.1080/07853890.2024.2425061)
Supplement: Table S2.docx [file IANN_A_2425061_SM0711.docx]

| **Table S2 Characteristics of females in the study population** | | | | | |
| --- | --- | --- | --- | --- | --- |
| **Variable** | **All subjects** | **Low Hb** | **Medium Hb** | **High Hb** | **p-value** |
| Hb (g/dL) | 133.6 (8.4) | 124.7 (3.5) | 133.4 (2.3) | 143.8 (4.7) | <.001 |
| Number of subjects (n) | 346 | 116 | 124 | 106 |  |
| Current smokers (n) | 68 | 24 | 21 | 23 | 0.649 |
| Insufficient sleep (n) | 116 | 36 | 45 | 35 | 0.636 |
| Sitting time ≥ 11 h/d (n) | 31 | 10 | 15 | 6 | 0.231 |
| BP medication (n) | 35 | 11 | 15 | 9 | 0.639 |
| Lipid medication (n) | 8 | 2 | 3 | 3 | 0.856 |
| Alcohol consumption (g/d) | 6.0 (7.9) | 5.7 (7.6) | 5.8 (7.8) | 6.5 (8.4) | 0.724 |
| MVPA (min/d) | 63.6 (30.0) | 66.6 (30.0) | 62.3 (32.2) | 61.7 (27.2) | 0.407 |
| Height (cm) | 165.5 (5.8) | 165.3 (6.4) | 165.3 (5.5) | 166.0 (5.4) | 0.554 |
| Weight (kg) | 69.8 (12.3) | 66.1 (8.8) | 68.9 (12.3) | 74.9 (13.9) | <.001 |
| BMI (kg/m^2^) | 25.5 (4.3) | 24.2 (3.1) | 25.2 (4.3) | 27.2 (4.9) | <.001 |
| WH ratio | 0.85 (0.05) | 0.84 (0.05) | 0.84 (0.05) | 0.86 (0.06) | 0.010 |
| Sbp mean (mmHg) | 118.7 (15.7) | 115.8 (13.4) | 118.6 (14.3) | 121.8 (18.8) | 0.017 |
| Dbp mean (mmHg) | 80.5 (10.3) | 77.8 (9.1) | 80.9 (10.0) | 83.1 (11.3) | <.001 |
| MAP (mmHg) | 93.2 (11.8) | 90.5 (10.2) | 93.5 (11.1) | 96.0 (13.4) | 0.002 |
| fB-glucose (mmol/L) | 5.2 (0.5) | 5.1 (0.4) | 5.1 (0.4) | 5.3 (0.5) | 0.004 |
| HOMA-IR | 1.50 (1.60) | 1.3 (1.0) | 1.5 (1.5) | 2.0 (2.0) | 0.014 |
| Total cholesterol (mmol/L) | 5.2 (0.8) | 5.1 (0.8) | 5.2 (0.8) | 5.3 (0.9) | 0.120 |
| HDL cholesterol (mmol/L) | 1.7 (0.4) | 1.74 (0.37) | 1.71 (0.37) | 1.62 (0.35) | 0.050 |
| LDL cholesterol (mmol/L) | 3.2 (0.8) | 3.1 (0.8) | 3.2 (0.7) | 3.4 (0.8) | 0.006 |
| Triglycerides (mmol/L) | 0.87 (0.46) | 0.78 (0.40) | 0.84 (0.57) | 1.01 (0.49) | 0.001 |
| Hematocrit (%) | 0.40 (0.02) | 0.38 (0.01) | 0.40 (0.01) | 0.43 (0.01) | <.001 |
| RBC count (10^12^/L) | 4.43 (0.30) | 4.2 (0.25) | 4.4 (0.20) | 4.7 (0.24) | <.001 |
| MCV (fL) | 90.5 (3.9) | 89.9 (4.2) | 90.4 (3.6) | 91.3 (3.6) | 0.021 |
| MCH (Pg) | 30.3 (1.5) | 29.8 (1.7) | 30.3 (1.3) | 30.8 (1.4) | <.001 |
| MCHC (g/dL) | 334.2 (6.9) | 331 (7) | 335 (6) | 337 (6) | <.001 |
| RDW (%) | 13.3 (0.8) | 13.5 (0.9) | 13.1 (0.7) | 13.2 (0.7) | <.001 |
| B-platelets (10^9^/L) | 264.5 (56.1) | 271 (60) | 260 (56) | 263 (51) | 0.286 |
| B-leucocytes (10^9^/L) | 5.37 (1.42) | 5.0 (1.3) | 5.3 (1.3) | 5.9 (1.6) | <.001 |
| B-neutrophils (10^9^/L) | 3.08 (1.10) | 2.8 (1.1) | 3.0 (0.9) | 3.4 (1.2) | <.001 |
| B-lymphocytes (10^9^/L) | 1.64 (0.47) | 1.5 (0.4) | 1.6 (0.5) | 1.8 (0.5) | <.001 |
| B-monocytes (10^9^/L) | 0.45 (0.14) | 0.43 (0.12) | 0.43 (0.13) | 0.48 (0.18) | 0.007 |
| B-eosinophils (10^9^/L) | 0.19 (0.13) | 0.17 (0.13) | 0.20 (0.12) | 0.19 (0.14) | 0.289 |
| B-basophils (10^9^/L) | 0.03 (0.02) | 0.03 (0.02) | 0.03 (0.02) | 0.03 (0.02) | 0.208 |
