# Supplementary figures and images for: Haemoglobin levels are associated with echocardiographic measures in a Finnish midlife population
Source: Ann Med. 2024 Dec 3;56(1):2425061. doi: 10.1080/07853890.2024.2425061 (PMC11616746; doi:10.1080/07853890.2024.2425061)

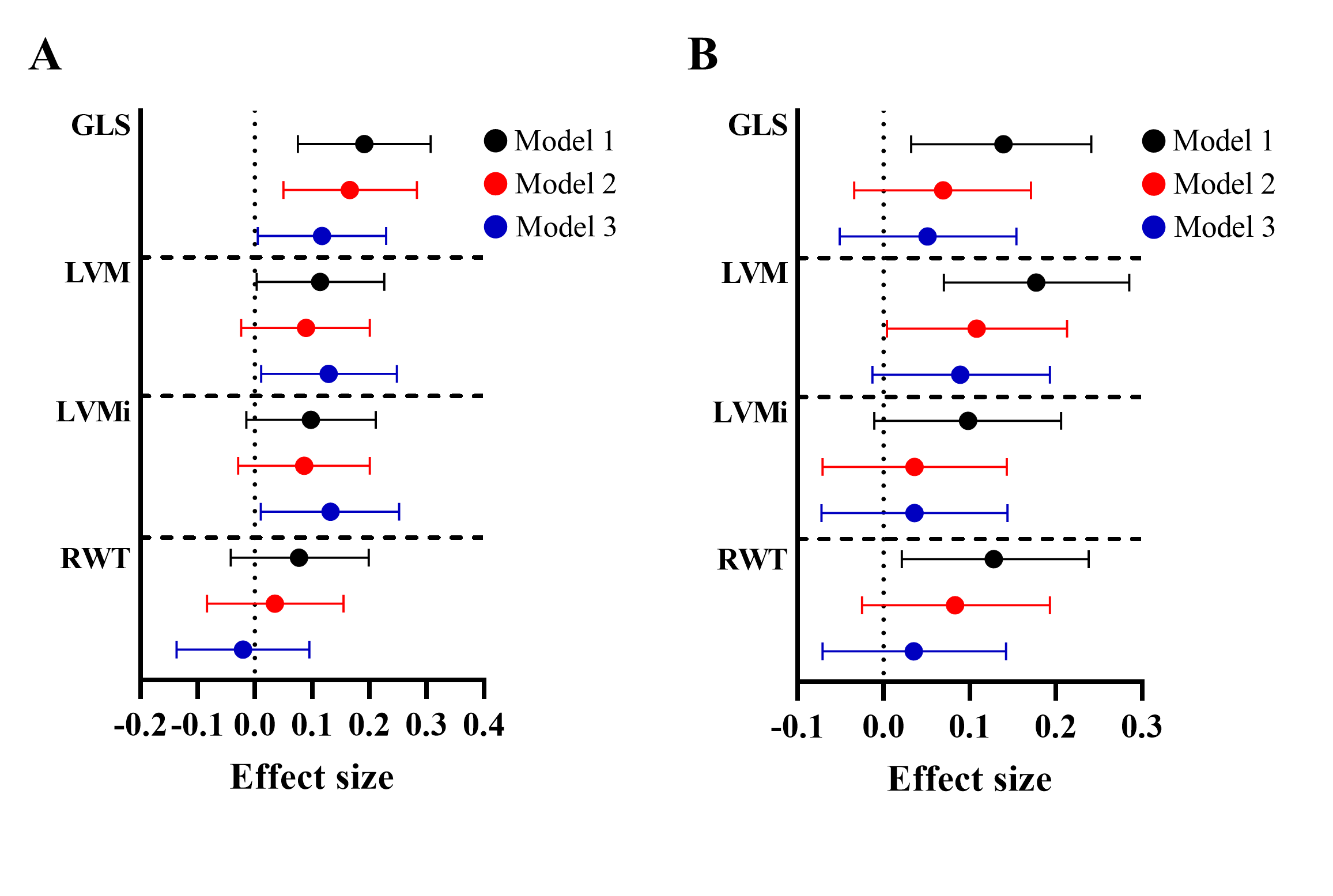

Supplement: S2.tif [file IANN_A_2425061_SM0710.tif]

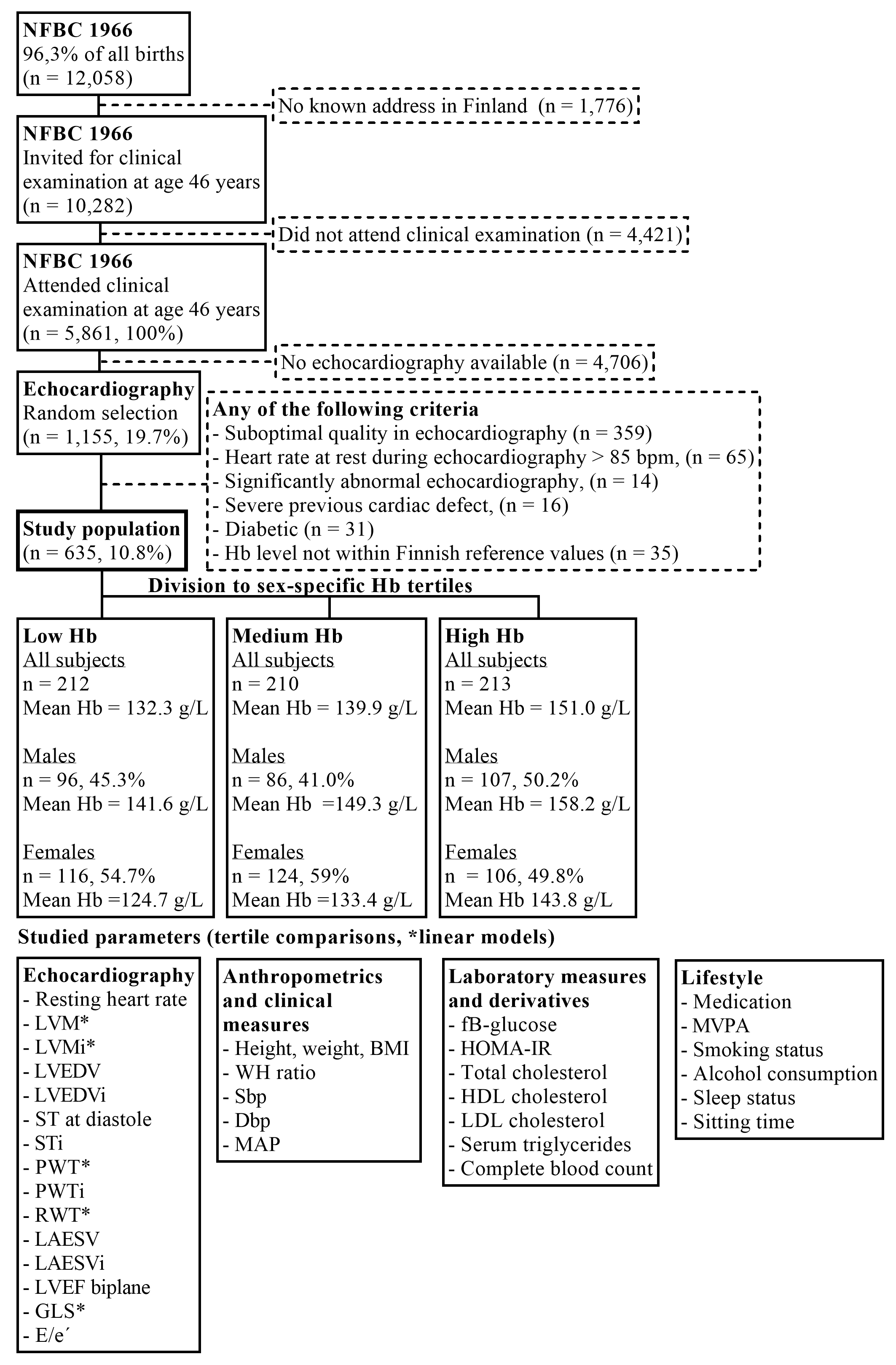

Supplement: S1.tif [file IANN_A_2425061_SM0708.tif]
