# Supplementary material for: Haemoglobin levels are associated with echocardiographic measures in a Finnish midlife population
Source: Ann Med. 2024 Dec 3;56(1):2425061. doi: 10.1080/07853890.2024.2425061 (PMC11616746; doi:10.1080/07853890.2024.2425061)
Supplement: Table S1.docx [file IANN_A_2425061_SM0709.docx]

| **Table S1 Characteristics of males in the study population** | | | | | |
| --- | --- | --- | --- | --- | --- |
| **Variable** | **All subjects** | **Low Hb** | **Medium Hb** | **High Hb** | ***P* value** |
| Hb (g/dL) | 150.0 (7.8) | 141.6 (3.5) | 149.3 (1.7) | 158.2 (4.7) | <.001 |
| Number of subjects (n) | 289 | 96 | 86 | 107 |  |
| Current smokers (n) | 72 | 23 | 23 | 26 | 0.883 |
| Insufficient sleep (n) | 75 | 29 | 22 | 24 | 0.463 |
| Sitting time ≥ 11 h/d (n) | 63 | 17 | 25 | 21 | 0.145 |
| Bp medication (n) | 38 | 17 | 10 | 11 | 0.260 |
| Lipid medication (n) | 5 | 0 | 3 | 2 | 0.195 |
| Alcohol consumption (g/d) | 16.1 (24.3) | 14.7 (23.2) | 17.5 (23.8) | 16.3 (25.8) | 0.746 |
| MVPA (min/d) | 81.2 (35.9) | 81.1 (34.9) | 81.6 (38.6) | 81.0 (35.1) | 0.992 |
| Height (cm) | 178.8 (6.1) | 178.9 (4.9) | 179.5 (6.7) | 178.1 (6.4) | 0.271 |
| Weight (kg) | 84.6 (11.4) | 82.1 (9.8) | 83.9 (11.3) | 87.5 (12.3) | 0.003 |
| BMI (kg/m^2^) | 26.5 (3.3) | 25.6 (2.8) | 26.0 (2.9) | 27.6 (3.7) | <.001 |
| WH ratio | 0.95 (0.05) | 0.94 (0.05) | 0.95 (0.06) | 0.96 (0.05) | 0.031 |
| Sbp mean (mmHg) | 128.7 (13.1) | 126.9 (11.0) | 127.7 (12.3) | 131.1 (15.0) | 0.052 |
| Dbp mean (mmHg) | 85.4 (9.0) | 83.8 (8.2) | 85.2 (8.8) | 87.0 (9.5) | 0.032 |
| MAP (mmHg) | 99.9 (9.9) | 98.2 (8.7) | 99.4 (9.5) | 101.7 (10.9) | 0.031 |
| fB-glucose (mmol/L) | 5.5 (0.5) | 5.4 (0.4) | 5.5 (0.4) | 5.6 (0.5) | 0.014 |
| HOMA-IR | 1.96 (1.43) | 1.8 (1.5) | 1.7 (1.5) | 2.3 (1.3) | 0.009 |
| Total cholesterol (mmol/L) | 5.5 (1.0) | 5.5 (1.0) | 5.6 (1.0) | 5.5 (0.9) | 0.742 |
| HDL cholesterol (mmol/L) | 1.4 (0.3) | 1.43 (0.28) | 1.40 (0.32) | 1.36 (0.28) | 0.243 |
| LDL cholesterol (mmol/L) | 3.7 (0.9) | 3.6 (0.9) | 3.7 (0.9) | 3.7 (0.8) | 0.432 |
| Triglycerides (mmol/L) | 1.22 (0.80) | 1.13 (0.60) | 1.28 (0.97) | 1.26 (0.88) | 0.228 |
| Hematocrit (%) | 0.44 (0.02) | 0.42 (0.01) | 0.44 (0.01) | 0.46 (0.02) | <.001 |
| RBC count (10^12^/L) | 4.94 (0.28) | 4.7 (0.2) | 4.9 (0.2) | 5.1 (0.2) | <.001 |
| MCV (fL) | 89.4 (3.2) | 89.3 (3.3) | 89.4 (3.2) | 89.5 (3.1) | 0.843 |
| MCH (Pg) | 30.5 (1.2) | 30.2 (1.2) | 30.4 (1.2) | 30.9 (1.1) | <.001 |
| MCHC (g/dL) | 341.1 (7.5) | 339 (7) | 340 (8) | 345 (7) | <.001 |
| RDW (%) | 13.1 (0.6) | 13.2 (0.6) | 13.1 (0.6) | 13.0 (0.6) | 0.147 |
| B-platelets (10^9^/L) | 232.6 (44.2) | 233 (40) | 231 (51) | 234 (44) | 0.933 |
| B-leucocytes (10^9^/L) | 5.25 (1.25) | 5.1 (1.2) | 5.2 (1.2) | 5.4 (1.3) | 0.251 |
| B-neutrophils (10^9^/L) | 2.76 (0.89) | 2.7 (0.8) | 2.7 (0.8) | 2.9 (1.0) | 0.138 |
| B-lymphocytes (10^9^/L) | 1.77 (0.49) | 1.7 (0.5) | 1.8 (0.5) | 1.8 (0.5) | 0.407 |
| B-monocytes (10^9^/L) | 0.49 (0.15) | 0.46 (0.13) | 0.49 (0.17) | 0.51 (0.15) | 0.118 |
| B-eosinophils (10^9^/L) | 0.21 (0.14) | 0.22 (0.13) | 0.21 (0.15) | 0.21 (0.13) | 0.735 |
| B-basophils (10^9^/L) | 0.03 (0.02) | 0.03 (0.02) | 0.03 (0.02) | 0.03 (0.02) | 0.842 |
