# Supplementary material for: Haemoglobin levels are associated with echocardiographic measures in a Finnish midlife population
Source: Ann Med. 2024 Dec 3;56(1):2425061. doi: 10.1080/07853890.2024.2425061 (PMC11616746; doi:10.1080/07853890.2024.2425061)
Supplement: Clean copy - GLS_manuscript_SUPPLEMENTAL_MATERIAL.docx [file IANN_A_2425061_SM0707.docx]

**Supplemental material**

**Hemoglobin levels are associated with echocardiographic measures in a Finnish midlife population**

Running head: Hemoglobin and cardiac function

Joona Tapio^1¤^, Tommi Grönlund ^2,3¤^, Kari Kaikkonen^2,3^, M. Juhani Junttila^2,3^, Mikko P. Tulppo^2,3*^, Peppi Koivunen^1*^

^1^University of Oulu, Biocenter Oulu and Faculty of Biochemistry and Molecular Medicine, Oulu Center for Cell-Matrix Research, Oulu, Northern Ostrobothnia, Finland, P.O. Box 5400, FIN-90014 Oulu, [joona.tapio@oulu.fi](mailto:joona.tapio@oulu.fi), [peppi.karppinen@oulu.fi](mailto:peppi.karppinen@oulu.fi)

^2^University of Oulu, Research Unit of Biomedicine and Internal Medicine, Oulu, Northern Ostrobothnia, Finland, P.O. Box 5000, FIN-90014 Oulu, [tommi.gronlund@oulu.fi](mailto:tommi.gronlund@oulu.fi), [kari.kaikkonen@oulu.fi](mailto:kari.kaikkonen@oulu.fi), [juhani.junttila@oulu.fi](mailto:juhani.junttila@oulu.fi), [mikko.tulppo@oulu.fi](mailto:mikko.tulppo@oulu.fi)

^3^Oulu University Hospital and University of Oulu, Medical Research Center Oulu, Oulu, Northern Ostrobothnia, Finland, P.O. Box 5000, FIN-90014 Oulu, [tommi.gronlund@oulu.fi](mailto:tommi.gronlund@oulu.fi), [kari.kaikkonen@oulu.fi](mailto:kari.kaikkonen@oulu.fi), [juhani.junttila@oulu.fi](mailto:juhani.junttila@oulu.fi), [mikko.tulppo@oulu.fi](mailto:mikko.tulppo@oulu.fi)

¤ Equal contribution

*To whom correspondence should be addressed:

Peppi Koivunen, University of Oulu, Biocenter Oulu and Faculty of Biochemistry and Molecular Medicine, Oulu Center for Cell-Matrix Research, Oulu, Northern Ostrobothnia, Finland, P.O. Box 5400, FIN-90014 Oulu, [peppi.karppinen@oulu.fi](mailto:peppi.karppinen@oulu.fi)

or Mikko P. Tulppo, University of Oulu, Research Unit of Biomedicine and Internal Medicine and Medical Research Center Oulu, Oulu, Northern Ostrobothnia, Finland, P.O. Box 5000, [mikko.tulppo@oulu.fi](mailto:mikko.tulppo@oulu.fi)

**Supplemental table legends**

**Table S1 Characteristics of males in the study population.**

The values indicate exact numbers of study subjects, mean (SD, standard deviation) or median (inter quartile range) for the variables used in the analysis. P is given for statistical comparison of low and High Hb tertiles. Number of participants (n) in the statistical analyses. Hb, hemoglobin; Bp, blood pressure; MVPA, moderate to vigorous physical activity; BMI, body mass index; WH, waist-hip; Sbp, systolic blood pressure; Dbp, diastolic blood pressure; MAP, mean arterial pressure; fB-glucose, fasting blood glucose; HOMA-IR, Homeostatic Model Assessment for Insulin Resistance; HDL, high-density lipoprotein; LDL, low-density lipoprotein; RBC, red blood cell; MCV, mean cellular volume; MCH, mean cellular hemoglobin; MCHC, mean cellular hemoglobin concentration; RDW, red cell distribution width; B-, blood.

**Table S2. Characteristics of females in the study population.**

The values indicate exact numbers of study subjects, mean (SD, standard deviation) or median (inter quartile range) for the variables used in the analysis. P is given for statistical comparison of low and High Hb tertiles. Number of participants (n) in the statistical analyses. Hb, hemoglobin; Bp, blood pressure; MVPA, moderate to vigorous physical activity; BMI, body mass index; WH, waist-hip; Sbp, systolic blood pressure; Dbp, diastolic blood pressure; MAP, mean arterial pressure; fB-glucose, fasting blood glucose; HOMA-IR, Homeostatic Model Assessment for Insulin Resistance; HDL, high-density lipoprotein; LDL, low-density lipoprotein; RBC, red blood cell; MCV, mean cellular volume; MCH, mean cellular hemoglobin; MCHC, mean cellular hemoglobin concentration; RDW, red cell distribution width; B-, blood.

**Table S3. Echocardiographic characteristics of males in the study population.**

The values indicate exact numbers of study subjects, mean (SD, standard deviation) or median (inter quartile range) for the variables used in the analysis. P is given for statistical comparison of low and High Hb tertiles. Number of participants (n) in the statistical analyses. LVM, left ventricular mass; i, index; LVEDV, left ventricular end-diastolic volume; ST, septal thickness, PWT, posterior wall thickness; RWT, relative wall thickness; LAESV, left atrial end-systolic volume; LVEF, left ventricular ejection fraction; GLS, global longitudinal strain; E/e’, ratio of early diastolic mitral inflow velocity to early diastolic mitral annulus velocity.

**Table S4. Echocardiography characteristics of females in the study population.**

The values indicate exact numbers of study subjects, mean (SD, standard deviation) or median (inter quartile range) for the variables used in the analysis. P is given for statistical comparison of low and High Hb tertiles. Number of participants (n) in the statistical analyses. LVM, left ventricular mass; i, index; LVEDV, left ventricular end-diastolic volume; ST, septal thickness, PWT, posterior wall thickness; RWT, relative wall thickness; LAESV, left atrial end-systolic volume; LVEF, left ventricular ejection fraction; GLS, global longitudinal strain; E/e’, ratio of early diastolic mitral inflow velocity to early diastolic mitral annulus velocity.

**Table S5.** **Effect sizes for association of Hb levels with echocardiographic parameters.**

Number of participants (n) in the statistical analyses. GLS, global longitudinal strain; LVM, left ventricular mass; i, index; RWT, relative wall thickness; B, unstandardized β; CIL, 95% confidence interval lower limit; CIU, 95% confidence interval upper limit. Model 1 is adjusted for sex. Model 2 is adjusted for Framingham risk factors (sex, body mass index, systolic blood pressure, smoking status, total cholesterol and HDL cholesterol). Model 3 is a stepwise linear regression adjusted for fasting glucose, diastolic blood pressure and waist-hip-ratio.

**Table S6. Effect sizes for association of Hb levels with echocardiographic parameters in males.**

Number of participants (n) in the statistical analyses. GLS, global longitudinal strain; LVM, left ventricular mass; i, index; RWT, relative wall thickness; B, unstandardized β; CIL, 95% confidence interval lower limit; CIU, 95% confidence interval upper limit. Model 1 is unadjusted. Model 2 is adjusted for Framingham risk factors (sex, body mass index, systolic blood pressure, smoking status, total cholesterol and HDL cholesterol). Model 3 is a stepwise linear regression adjusted for fasting glucose, diastolic blood pressure and waist-hip-ratio

**Table S7. Effect sizes for association of Hb levels with echocardiographic parameters in females.** Number of participants (n) in the statistical analyses. GLS, global longitudinal strain; LVM, left ventricular mass; i, index; RWT, relative wall thickness; B, unstandardized β; CIL, 95% confidence interval lower limit; CIU, 95% confidence interval upper limit. Model 1 is unadjusted. Model 2 is adjusted for Framingham risk factors (sex, body mass index, systolic blood pressure, smoking status, total cholesterol and HDL cholesterol). Model 3 is a stepwise linear regression adjusted for fasting glucose, diastolic blood pressure and waist-hip-ratio

**Supplemental figure legends**

**Figure S1. Flow-chart representing the study population and analyses done.** Number of participants (n) in the statistical analyses. NFBC, Northern Finland Birth Cohort; Hb, hemoglobin; LVM, left ventricular mass; LVMi, LVM index; LVEDV, left ventricular end-diastolic volume; ST, septal thickness; PWT, posterior wall thickness; RWT, relative wall thickness; LA, left atrial; ESV, end-systolic volume; LVEF, left ventricular ejection fraction; GLS, global longitudinal strain; E/e’, ratio of early diastolic mitral inflow velocity to early diastolic mitral annulus velocity; BMI, body mass index; MAP, mean arterial pressure; HDL, high density lipoprotein; LDL, low density lipoprotein; HOMA-IR, Homeostatic Model Assessment for Insulin Resistance.

**Figure S2. Sex-specific effect sizes for association of Hb levels with echocardiographic parameters**. A = males. B = females. Forest plot representing the effect sizes and their 95% confidence intervals for selected echocardiographic parameters. GLS, global longitudinal strain; LVM, left ventricular mass; i, index; RWT, relative wall thickness. Model 1 (black) is unadjusted. Model 2 (red) is adjusted for Framingham risk factors (sex, body mass index, systolic blood pressure, smoking status, total cholesterol and HDL cholesterol). Model 3 (blue) is a stepwise linear regression adjusted for fasting glucose, diastolic blood pressure and waist-hip-ratio.
