# Supplementary material for: Haemoglobin levels are associated with echocardiographic measures in a Finnish midlife population
Source: Ann Med. 2024 Dec 3;56(1):2425061. doi: 10.1080/07853890.2024.2425061 (PMC11616746; doi:10.1080/07853890.2024.2425061)
Supplement: Table S5.docx [file IANN_A_2425061_SM0706.docx]

| **Table S5** **Effect sizes for association of Hb levels with echocardiographic parameters** | | | | | | |
| --- | --- | --- | --- | --- | --- | --- |
|  |  | **n** | **B** | **CIL** | **CIU** | ***P* value** |
| **GLS** | **Model 1** | 635 | 0.221 | 0.124 | 0.317 | < 0.001 |
|  | **Model 2** | 635 | 0.140 | 0.043 | 0.238 | 0.005 |
|  | **Model 3** | 635 | 0.153 | 0.071 | 0.235 | < 0.001 |
| **LVM** | **Model 1** | 635 | 0.204 | 0.115 | 0.292 | < 0.001 |
|  | **Model 2** | 635 | 0.102 | 0.015 | 0.188 | 0.021 |
|  | **Model 3** | 635 | 0.272 | 0.193 | 0.350 | < 0.001 |
| **LVMi** | **Model 1** | 635 | 0.165 | 0.067 | 0.264 | < 0.001 |
|  | **Model 2** | 635 | 0.068 | -0.030 | 0.166 | 0.175 |
|  | **Model 3** | 635 | 0.229 | 0.141 | 0.316 | < 0.001 |
| **RWT** | **Model 1** | 635 | 0.142 | 0.029 | 0.247 | 0.009 |
|  | **Model 2** | 635 | 0.082 | -0.029 | 0.190 | 0.151 |
|  | **Model 3** | 635 | 0.033 | -0.078 | 0.142 | 0.532 |
